# Plasma cell vulvitis: A systematic review

**DOI:** 10.1016/j.ijwd.2021.04.005

**Published:** 2021-05-04

**Authors:** Samantha Sattler, Ashley N. Elsensohn, Melissa M. Mauskar, Christina N. Kraus

**Affiliations:** a Albany Medical College, Albany, New York; b University of California, San Diego, San Diego, California; c University of Texas Southwestern Medical Center, Department of Dermatology, Dallas, Texas; d University of California, Irvine, Irvine, California

**Keywords:** Plasma cell vulvitis, Vulvar disease, Plasma cell mucositis, Zoon's vulvitis

## Abstract

**Background:**

Plasma cell vulvitis (PCV) is an inflammatory vulvar dermatosis that is not well characterized. Diagnosis is often delayed, and the condition can be refractory to treatment. To date, there are no systematic reviews on this topic.

**Objective:**

This study aimed to provide a systematic review of PCV, including epidemiologic, clinical, and histopathologic findings, as well as associated comorbidities and treatment options.

**Methods:**

A primary literature search was conducted using the PubMed, Ovid Medline, Cochrane, and CINAHL databases.

**Results:**

Fifty-three publications with 196 patients (mean age: 55.3 ± 14.5 years) were included. The majority of studies were case reports and case series. Common symptoms included burning/stinging (52%), dyspareunia (44%), and pruritus (41%). Common findings included erythema (84%), glistening/shiny appearance (29%), well-demarcated lesions (25%), and erosions (22%). Common anatomic sites were the labia minora (45%), introitus (31%), and periurethral (19%). Fifty-three percent of patients had a solitary lesion. Common histologic findings were a predominant plasma cell infiltrate (88%), presence of other inflammatory cells (55%), hemosiderin/siderophages (46%), and epidermal atrophy (43%). Topical corticosteroids (64%) and tacrolimus ointment (13%) were the most frequent treatment modalities. In most reports, previous treatments were tried, and there was a diagnostic delay.

**Conclusion:**

PCV is likely underrecognized and should be considered in patients with erythema of the mucous and modified mucous membranes, symptoms of burning or stinging, and a predominant plasma cell infiltrate on histopathology. First-line therapy should begin with high-potency topical corticosteroids, with the most evidence for clobetasol 0.05% or tacrolimus 0.1% ointment. Prospective studies are needed to further characterize this condition and to develop treatment guidelines.

## Introduction

**Fig. 1:**
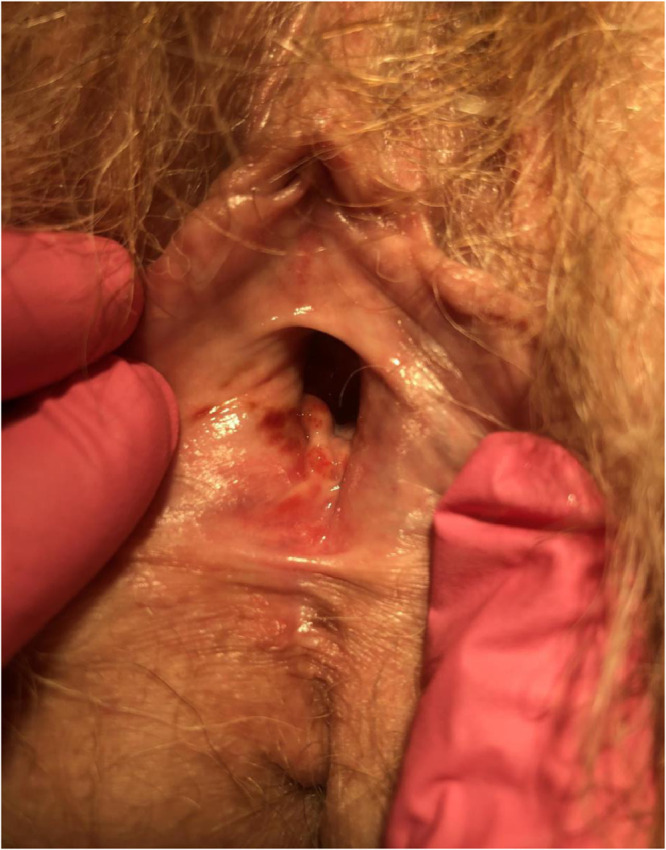
Clinical image of plasma cell vulvitis.

**Fig. 2:**
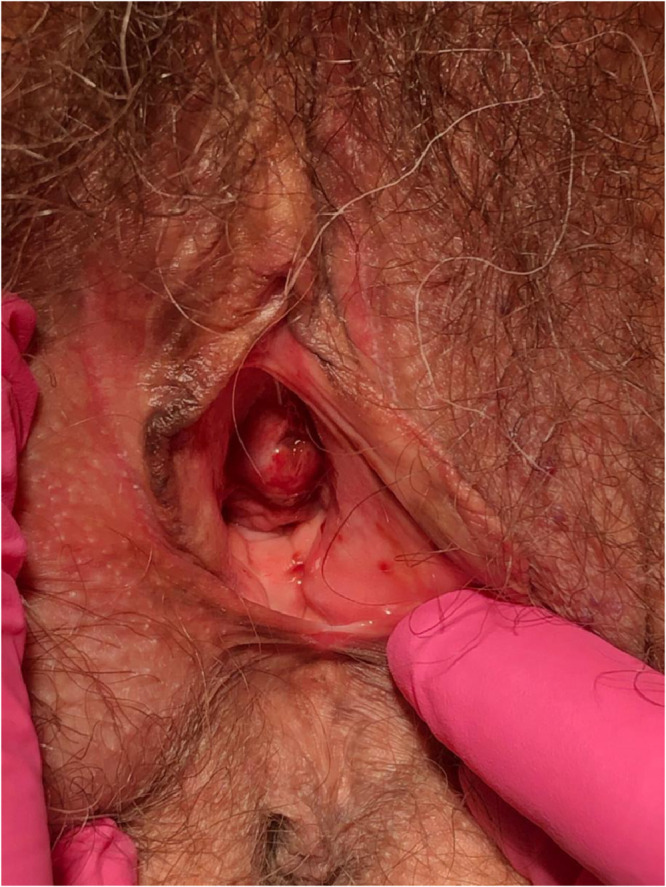
Clinical image of plasma cell vulvitis.

Plasma cell vulvitis (PCV), previously Zoon's vulvitis or vulvitis circumscripta plasmacellularis, is an uncommon idiopathic dermatosis originally described in 1952 (Zoon, 1952). PCV is thought to exist on a spectrum of plasma cell mucositis, which can involve the oral mucosa (buccal mucosa, lips, palate, gingiva, tongue, epiglottis, larynx) and glans penis (plasma cell balanitis; Brix et al., 2010). The characteristic clinical findings are bright red to cayenne pepper or orange-hued well-demarcated macules/patches involving the vulvar vestibule, periurethral area, and labia minora (Fig. 1, Fig. 2). The condition may be asymptomatic but commonly presents with symptoms of burning, stinging, pruritus, and pain (Bharatia et al., 2015).

**Fig. 3:**
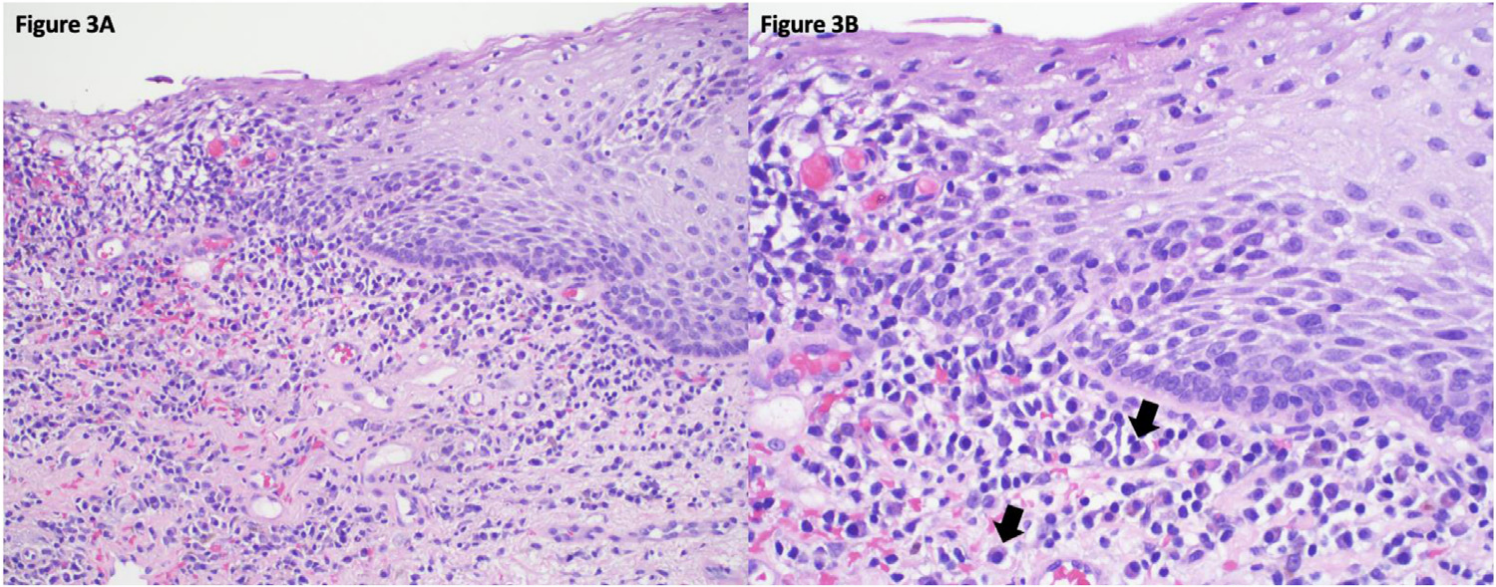
Histologic images of plasma cell vulvitis.

Its etiology is unknown, but PCV has been reported to occur in patients with coexistent autoimmune conditions, suggesting an autoimmune etiology. Additionally, hormonal, infectious, and irritant factors have been implicated in the pathogenesis of PCV (Bharatia et al., 2015; Celik et al., 2012). Histopathologic findings (Figs. 3A and B) include an inflammatory infiltrate consisting of predominantly polyclonal plasma cells, along with lozenge- or diamond-shaped keratinocytes and erythrocyte extravasation or hemosiderin deposition (Virgili et al., 2005). Information on the clinical course of PCV is limited, primarily owing to few large-scale observational studies and the rarity of this condition.

The aim of this study is to perform a systematic review on cases of PCV in the literature to summarize the epidemiologic, clinical, and histologic findings and to identify associated comorbidities and treatment options for patients with PCV.

## Methods

### Literature search

**Fig. 4:**
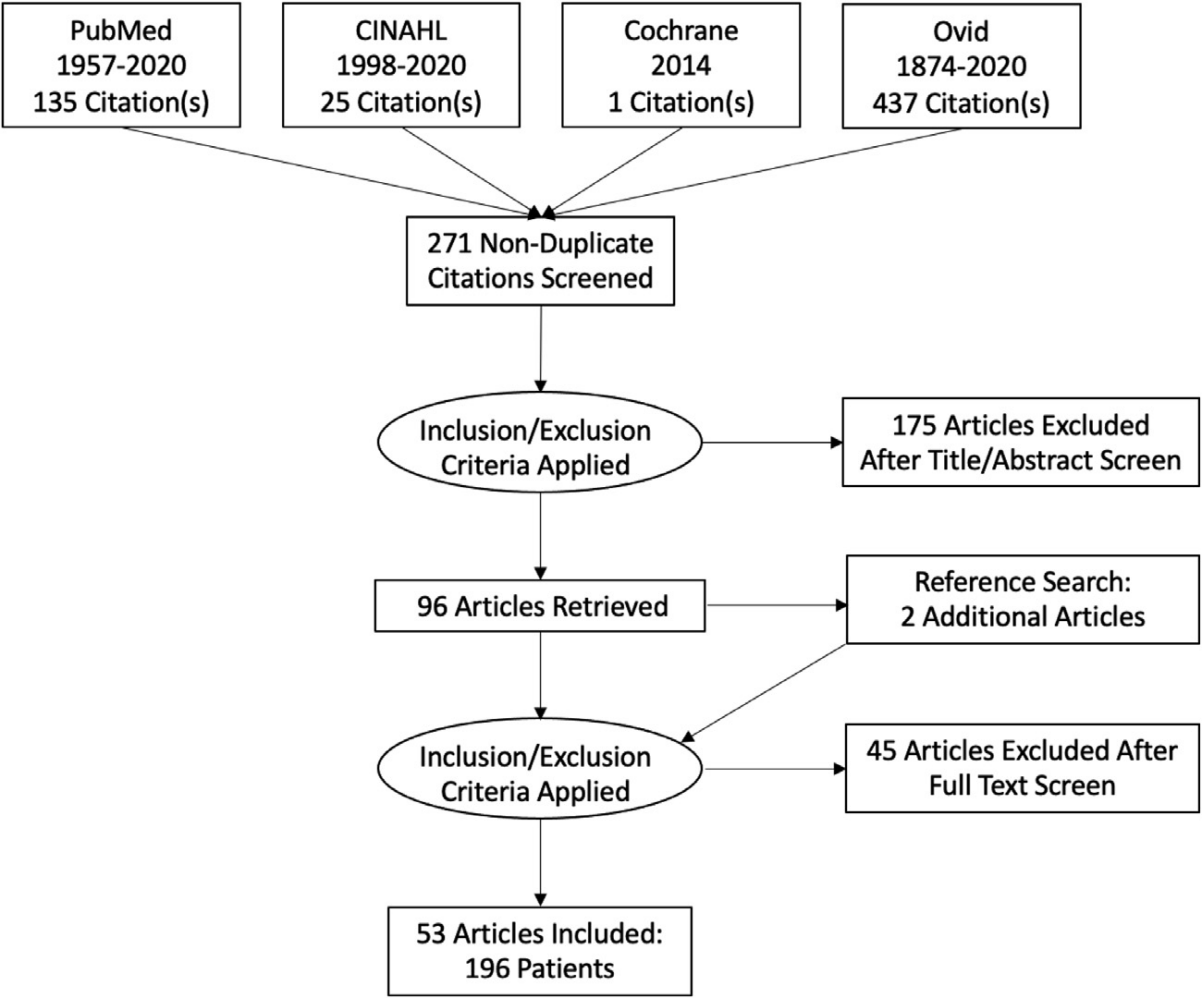
Preferred Reporting Items for Systematic Reviews and Meta-Analyses diagram.

This study was done in accordance with the Preferred Reporting Items for Systematic Reviews and Meta-Analyses. A primary literature search was conducted using PubMed, Ovid Medline, Cochrane, and CINAHL on October 20, 2020. Two authors independently searched and cross-checked with the following search terms: “zoon's vulvitis,” “zoon vulvitis,” “plasma cell vulvitis,” and “vulvitis circumscripta plasmacellularis.” Reference lists of included articles were further screened for additional publications not identified through the initial search strategy (Fig. 4).

### Article selection

Articles published in English from all years were considered for eligibility. Articles were excluded based on title, abstract, or both if there was no clear indication they were investigating PCV. Reviews were included in our initial search to avoid omitting any novel cases in papers labeled as “review.” Subsequently identified studies were subjected to a full-text review.

### Data extraction

Included studies were summarized using a data extraction form where the following variables were extracted: age, ethnicity, location, clinical findings, symptoms, histopathologic findings, treatment modality, prior treatment modalities, and comorbidities.

## Results

### Literature review

The initial literature search yielded 598 manuscripts. Overall, 271 were nonduplicate articles. A total of 175 articles were excluded based on their title and/or abstract, including 169 that were not related to PCV and six that were not available in the English language. Forty-four additional articles were excluded because they did not include a presentation of PCV or an evaluation of an existing PCV case. One article that reported eight cases of “Zoon-like” findings in papular colpitis was excluded because the authors included these cases as clinically suggestive of Zoon's vulvitis but did not diagnose these cases as Zoon's (Van der Meijden and Ewing, 2011). Two additional articles were identified based on a search of article references. Two papers labeled as reviews underwent data abstraction: a histopathologic review of cases at a single institution (Chan and Zimarowski, 2015) and a case report including a literature review with only information from the case extrapolated (Pourzan and Laird-Fick, 2019).

Ultimately, 53 manuscripts met the eligibility criteria and were included in this qualitative analysis (Fig. 4). All articles were published between 1981 and 2020 (Ajibade and Hirsi-Farah, 2019; Ajibona and Brown, 2005; Albers et al., 2000; Baba et al., 2017; Bharatia et al., 2015; Bhaumik et al., 2006; Botros et al., 2006; Brix et al., 2010; Celik et al., 2012; Chan and Zimarowski, 2015; Corazza et al., 2018; Damiani et al., 2017; David and Massey, 2003; Davis et al., 1983; Dos Reis et al., 2013; Ee et al., 2003; Elghobashy et al., 2020; Fernandez-Acenero and Cordova, 2010; Frega et al., 2006; Greco et al., 2016; Gunter and Golitz, 2005; Gurumurthy et al., 2010; Hautmann et al., 1994; Hindle et al., 2006; Jaimes Suarez et al., 2017; Kavanagh et al., 1993; Kuniyuki et al., 1998; McCreedy and Melski, 1990; Morioka et al., 1988; Nedwich and Chong, 1987; Neri et al., 1995; Page et al., 2015; Pourzan and Laird-Fick, 2019; Powers et al., 2015; Robinson et al., 1998; Mitchell et al., 2020; Salopek and Siminoski, 1996; Santonja et al., 2019; Scurry et al., 1993; Sharma et al., 2019; Solt et al., 2004; Souteyrand et al., 1981; Thomson et al., 2007; Toeima et al., 2011; Van der Meijden and Ewing, 2011; van Kessel et al., 2010; Virgili et al., 2004; 2005; 2008; 2015a; 2015b; Woodruff et al., 1989; Yoganathan et al., 1994).

The eligible manuscripts consisted of case series, case reports, retrospective studies, and two prospective observational studies, comprising a total of 196 patients with PCV (Fig. 4). The majority of manuscripts were published in dermatology journals (n = 24 of 53 [45%]). Gynecology journals were the second most common journal type (n = 17 of 53 [32%]), followed by pathology journals (n = 4 of 53 [7.5%]), other genitourinary journals (n = 4 of 53 [7.5%]), infectious disease journals (n = 3 of 53 [6%]), and other journals (n = 1 of 53 [2%]).

### Clinical presentation

The age distribution ranged from 8 to 84 years, with a mean of 55.3 ± 14.5 years. There was one case reported in a pediatric patient (8 years of age; Albers et al., 2000). Due to the lack of ethnicity data included, we could not determine any ethnic distribution for PCV. In two of the largest studies, there was a mean delay in diagnosis of 4.7 years (Virgili et al., 2015a; 2015b). Our analysis revealed a wide range of diagnostic delays (excluding the two Virgili studies mentioned), from 6 weeks to 10 years, with a median of 12 months. Symptomatology was presented in 49 publications, but one study was excluded when tabulating symptom frequency because the percent of patients with symptoms was not specified (Virgili et al., 2008). The 49 publications included 155 patients (79%), and the most frequent symptom was a burning or stinging sensation (n = 80 [52%]), followed by dyspareunia (n = 68 [44%]), pruritus (n = 63 [41%]), and other pain/tenderness (n = 30 [19%]). Other symptoms included dysuria (n = 10), vaginal discharge (n = 9), irritation (n = 5), bleeding/spotting (n = 4), dryness (n = 3), and incontinence (n = 1). Eight patients (5%) were asymptomatic.

Clinical presentation was described in 49 publications including 159 patients. Studies without clinical findings included a study on PCV dermoscopy and two studies on PCV histopathology (Brix et al., 2010; Chan and Zimarowski, 2015; Corazza et al., 2018). Two of the largest scale studies reported clinical findings to be chronic, well-circumscribed, erythematous, glistening patches with a faint orange hue, while erosions and cayenne pepper spots were supportive features (total of 60 patients), but individual patient findings could not be extrapolated from these studies (Virgili et al., 2015a; 2015b). Another study of 20 patients reported that the findings were typical lesions but did not elaborate further (Virgili et al., 2005).

Of the remaining 46 publications with clinical findings described (total of 79 patients), erythema was the most common (n = 66 [84%]), with seven cases (10%) noting the findings as “bright red.” A glistening, glazed, or shiny appearance was noted in 23 cases (29%), and 20 cases (25%) were characterized as well demarcated or well circumscribed. The next common finding was erosions in 17 cases (22%), followed by an orange hue in 11 cases (14%). Less commonly reported findings included ulceration (10% or 13%) and brown hue (5% or 6%). Of the 64 patients for whom solitary or multiple lesions could be determined, a slight majority of cases were found to be solitary (34% or 53%).

Anatomical distribution of PCV was reported in 49 publications (159 patients), and the most common site was the labia minora in 71 patients (45%), followed by introitus (50% or 31%), periurethral area (30% or 19%), vestibule (24% or 15%), and posterior fourchette (21% or 13%). The clitoris and clitoral hood were involved less frequently (15% or 9%), and the labia majora was involved in only 6 cases (4%). The perineum was involved in 2 cases (1%). Although not consistently specified, we extrapolated the number of lesions from 132 patient cases; the slight majority of patients had one site of involvement (70% or 53%), and 62 patients (47%) had more than one site involved.

### Histologic characteristics

A diagnosis was made based on histopathologic findings in all publications, but 42 publications (consisting of 84 patients) reported histologic findings that could be tabulated (Table 1). Of these, two histopathologic studies were included in our analyses (Brix et al., 2010; Virgili et al., 2005). The remaining 40 publications consisted of case reports and cases series.

**Table 1:** Histopathologic findings (N = 84)

Histopathologic finding	n	%
Predominantly plasma cell infiltrate	74	88
Other inflammatory cells (mast cells, lymphocytes, eosinophils, neutrophils)	46	55
Hemosiderin/siderophages	39	46
Epidermal atrophy	36	43
Dilated blood vessels	31	37
Erythrocyte extravasation	26	31
Dense plasma cell infiltrate*	21	25
Lozenge-shaped or diamond-shaped keratinocytes	18	21
Spongiosis	13	15
Increased number of blood vessels	10	12
Russel bodies	7	8
Polyclonality	3	4
Mucinous metaplasia	3	4
Antitreponemal or spirochete staining	2	2
Ulceration	2	2
Parakeratosis	1	1
Sparse plasma cells	0	0
Presence of lymphoid follicles	0	0
Fibrosis	0	0

⁎ Plasma cell infiltrate was deemed dense if specified in the publication as dense or included ≥50% plasma cells. Sum of treatments does not equal total patients due to cases with multiple findings

The most common finding was a predominantly plasma cell inflammatory infiltrate (73 cases [88%]), followed by other inflammatory cells (46 cases [55%]). A dense plasma cell infiltrate was considered if the report stated “dense” or included ≥50% plasma cells, which was found in 21 cases (25%). Hemosiderin/siderophages and erythrocyte extravasation was found in 39 (46%) and 26 (31%) of cases, respectively. Dilated blood vessels and increased number of blood vessels were reported in 31 (37%) and 10 (12%) cases, respectively. Epidermal atrophy was observed in 36 cases (43%). Lozenge- or diamond-shaped keratinocytes were seen in 18 cases (21%) and spongiosis in 13 cases (15%). Russel bodies (n = 7), ulceration (n = 2), mucinous metaplasia (n = 3), and parakeratosis (n = 1) were less frequently reported findings.

### Treatment

Forty-six studies consisting of 94 patients (47%) evaluated treatment modalities (Table 2). Other than a retrospective study of 24 patients that compared topical corticosteroids with a topical fusidic acid/betamethasone combination and topical tacrolimus 0.1% ointment (Virgili et al., 2015a), publications on treatment were limited to case reports and case series. The most common treatment modality was topical corticosteroids (60% or 64%), either alone (44% or 47%) or in combination with another agent (16% or 17%). Clobetasol was the most frequently used topical steroid (n = 15 of 44 [34%] of topical steroids used; Table 3). Twelve cases did not specify the type of topical steroid used, and four cases remarked that it was a mid-potency to ultrapotent topical steroid. Hydrocortisone, either topically or as an intravaginal suppository, was the next most frequently used topical steroid (n =6 of 44 [13%]).

**Table 2:** Treatment modalities (N = 94)

Topical			Systemic		
Treatment	n	%	Treatment	n	%
**Topical corticosteroids**	44	47	**Oral antibiotics**	5	5
**Fusidic acid 2% and betamethasone valerate 0.1% cream**	14	15	**Oral corticosteroids**	2	2
**Tacrolimus 0.1% ointment**	10	11⁎	**Oral estrogen**	2	2
**Tacrolimus 0.03% ointment**	2	2	**Oral progesterone**	2	2
**Imiquimod**	8	9	**Etretinate**	2	2
**Estrogen**	7	7	**Interferon**	2	2
**Misoprostol**	3	3	**Acyclovir**	1	1
**Vaginal estradiol/hydrocortisone/clindamycin**	2	2			
**Fusidic acid**	1	1			
**Antifungal not otherwise specified**	1	1			
**Cyclosporine**	1	1			
**Erythromycin**	1	1			
**Gentamicin**	1	1			

Other					
Treatment	n	%			

**Excision/partial vulvectomy**	3	3			
**CO_2_ laser therapy**	2	2			
**Platelet-rich plasma**	1	1			
**Fulguration**	1	1			

⁎ Sum of treatments does not equal total patients due to cases with multiple treatments.

**Table 3:** Topical corticosteroids used by potency, formulation, and vehicle (N = 44)

Topical corticosteroid used	n
Clobetasol 0.05% ointment	7
Clobetasol 0.05% cream	7
Clobetasol (unknown strength and vehicle)	1
Hydrocortisone 1% cream	4
Betamethasone dipropionate 0.5% cream/ointment	3
Betamethasone valerate 0.05% ointment	1
Betamethasone valerate 0.1% (unknown vehicle)	1
Fluocinolone acetonide 0.025% cream	1
Mometasone 0.1% cream	1
Hydrocortisone suppository (unknown strength)	1
Hydrocortisone (unknown strength and vehicle)	1
“Potent” topical steroid	4
Unknown topical steroid	12

A combination of fusidic acid 2% and betamethasone valerate 0.1% cream was the second most frequent treatment modality (n = 14 of 88 [16%]), as a retrospective cohort study compared this combination with clobetasol propionate 0.05% and tacrolimus 0.1% ointment (Virgili et al., 2015a). Tacrolimus 0.1% ointment was sed in 10 of 94 cases (11%) with improvement. Of these 10 cases, seven had used topical steroids without improvement. Tacrolimus 0.03% ointment was used in 2 of 94 cases but was discontinued in both cases due to local irritation (Gunter and Golitz, 2005). Imiquimod was used in 8 of 94 cases (9%).

Systemic therapy was less common, with oral antibiotics (minocycline, doxycycline, or a cephalosporin) used most commonly in 5 of 94 cases (5%). Infrequently, the following systemic therapies were used: corticosteroids, estrogen, progesterone, etretinate, interferon, and acyclovir. Other treatment modalities used included partial vulvectomy, CO_2_ laser therapy, platelet-rich plasma, and fulguration.

### Disease associations

Disease associations were rarely reported, and we did not identify any reports of PCV progressing to differentiated vulvar intraepithelial neoplasia. We found three cases of mucinous metaplasia developing into PCV (Santonja et al., 2019; Thomson et al., 2007). There were six patients with coexisting autoimmune conditions, including cutaneous lupus, autoimmune thyroiditis, rheumatoid arthritis, and adrenal insufficiency (Gunter and Golitz, 2005; Hindle et al., 2006; Kavanagh et al., 1993; Salopek and Siminoski, 1006; Scurry et al., 1993; Virgili et al., 2005). There were two cases of concomitant vulvar lichen sclerosus and one case of oral lichen planus (Powers et al., 2015; Toeima et al., 2011; van Kessel et al., 2010). Other concomitant conditions included human papillomavirus (n = 3), herpes simplex virus (n = 2), candidiasis (n = 2), HIV (n = 2), atopy/asthma (n = 2), and Sweet's syndrome (n = 1).

## Discussion

In this review, we summarize the clinical and histological findings, as well as treatment modalities, for patients with PCV. Although the clinical features of PCV are quite distinctive, this is an uncommon inflammatory vulvar dermatosis, and diagnosis is often delayed due to providers’ unfamiliarity with this condition. There is a paucity of information on PCV, and of the studies we identified, the majority of patients experienced a diagnostic delay and had failed multiple topical and systemic treatments prior to receiving a diagnosis of PCV.

### Clinical presentation

We found that a majority of patients presented with an erythematous solitary lesion, which is commonly well demarcated and has a shiny or glazed appearance. The most frequent site is the labia minora, followed by the introitus and periurethral areas. The labia majora is almost never involved because PCV primarily affects mucosal surfaces. The labia majora consists of keratinized, hair-bearing and non-hair-bearing epithelium, while the labia minora and introitus consist of modified mucous membranes.

### Histopathologic findings

Histologically, PCV is characterized by an inflammatory infiltrate consisting predominantly of plasma cells. A histopathologic study of 18 PCV cases found that the percentage of plasma cells appears to be the most important parameter when diagnosing PCV and that when plasma cells are ≥50%, this is sufficient for diagnosis (Virgili et al., 2005). All studies in our review relied on histopathology for diagnosis of PCV, but not all publications reported histopathologic findings. The most frequently reported finding in our analysis was a predominantly plasma cell infiltrate in 92% of cases.

Larger scale studies are necessary on clinicopathologic correlations, such as comparing chronicity with histopathologic features. The goal would be to find more reproducible and earlier markers to help establish a diagnosis. Three cases reported mucinous metaplasia of the vulva, which has been speculated to develop in the setting of chronic inflammation. Because definitive diagnosis of PCV requires tissue biopsy, it is important to differentiate the histologic findings from other inflammatory conditions, such as erosive vulvovaginal lichen planus, differentiated vulvar intraepithelial neoplasia, and infectious etiologies (syphilis). A histopathologic review of vulvar dermatology cases, including 15 cases of PCV, identified basal keratinocyte crowding as a new finding in the majority of cases (86.7%; Chan and Zimarowski, 2015). This finding was not reported in any additional studies in our analysis but may be a useful criterion to diagnose PCV going forward and should be evaluated in future histopathologic studies.

### Therapeutic modalities

The most common treatment modalities for PCV consisted of topical corticosteroids, with clobetasol being the most frequently used topical steroid. The largest comparative study we analyzed, by Virgili et al. (2015a) and based in Italy, is a retrospective cohort of patients, comparing fusidic acid 2% in combination with betamethasone valerate 0.1% cream to clobetasol propionate 0.05% ointment and tacrolimus 0.1% ointment. The authors found symptomatic improvement in patients with both treatment regimens. Symptom relief was observed to be greater with topical clobetasol and tacrolimus 0.1% ointment compared with fusidic acid/betamethasone. However, there were no statistically significant differences in clinical response among the treatment groups at any timepoint. The authors also found a mean delay in diagnosis of almost 5 years and that about 80% of patients had been treated previously.

Overall, topical mid- to ultrapotent corticosteroids, followed by tacrolimus 0.1% ointment, are the most frequently reported treatment modalities with success. However, information regarding duration of therapy, treatment time to resolution or recurrence, and measure of treatment efficacy is limited and variable. Other treatments have been reported, but evidence is limited and thus we cannot formulate any recommendations for the following treatments: topical imiquimod, topical misoprostol, topical and systemic antibacterial or antifungal therapies, systemic etretinate, cyclosporine, interferon, and procedural-based therapies (e.g., partial vulvectomy, CO_2_ laser therapy, platelet-rich plasma, and fulguration.

### Disease associations/concomitant dermatoses

Because contact allergy has been demonstrated in multiple vulvar dermatoses, including lichen sclerosus, development of contact dermatitis in seven patients with PCV was evaluated in a study that found a low frequency of contact sensitivity in these patients (Virgili et al., 2004). The authors concluded that patch testing is not necessary for patients with PCV unless patients experience symptoms of burning/itching after the application of topicals. Further data on concomitant irritant or allergic contact dermatitis in these patients may be useful in identifying whether an allergen or irritant plays a role in development or perpetuation of PCV.

Disease associations were rarely reported but included autoimmune conditions (e.g., cutaneous lupus, autoimmune thyroiditis, rheumatoid arthritis, adrenal insufficiency). There were two cases of concomitant vulvar lichen sclerosus and one case of oral lichen planus.

### Study limitations

The main limitation of this review is study design: Supporting evidence is derived predominantly from case reports and case studies. Our study is limited by interstudy variability and heterogeneity of results. Many studies were missing variables that are useful for providing diagnostic or therapeutic recommendations.

## Conclusion

In this review, we characterized the epidemiologic, clinical, and histologic features of PCV and summarized reported therapeutic modalities. Although the anatomic location and clinical appearance of PCV are characteristic, this is an underrecognized condition and diagnosis is often delayed. First-line treatment should consist of ultrapotent topical corticosteroids ointment or topical tacrolimus 0.1% ointment.

Summarizing the data of reported cases and studies provides further information on the characteristics of this uncommon disease and portends an earlier diagnosis of PCV. The diagnosis of PCV is still debated, with some considering PCV a variant of lichen planus and others considering PCV to be a chronic inflammatory sign of other vulvar dermatoses rather than a distinct entity. We view PCV as a distinct clinical entity and one that needs further characterization. Vulvar dermatoses often overlap; however, solitary red-orange patches on the modified mucous membranes warrant biopsy and clinicopathologic correlation to diagnose PCV. The symptoms of PCV may improve with topical therapy, but signs of the disease can be quite refractory, leading to decreased quality of life for these patients. Prospective studies are needed to further characterize this condition to better understand its pathogenesis and histopathologic features associated with disease chronicity and ultimately to develop treatment guidelines.

## References

[bib0001] Ajibade F., Hirsi-Farah S. (2019). Zoon's vulvitis and importance of histology in diagnosis of benign vulvar lesions in outpatient setting. Eur J Obstet Gynecol Reprod Biol.

[bib0002] Ajibona O., Brown L. (2005). Apareunia in a patient with Zoon's vulvitis. J Obstet Gynecol.

[bib0003] Albers S.E., Taylor G., Huyer D., Oliver G., Krafchik B.R. (2000). Vulvitis circumscripta plasmacellularis mimicking child abuse. J Am Acad Dermatol.

[bib0004] Baba Y., Umegaki-Arao N., Kimura Y. (2017). Successful treatment of intractable vulvitis circumscripta plasmacellularis via combination therapy with topical tacrolimus and tetracycline. J Dermatol.

[bib0005] Bharatia P.R., Pradhan A.M., Zawar V.P. (2015). Plasma cell vulvitis. Indian J Sex Transm Dis AIDS.

[bib0006] Bhaumik J. (2006). Vulvitis circumscripta plasmacellularis: Success with a modified treatment regimen using imiquimod. J Obstet Gynaecol (Lahore).

[bib0007] Botros S.M., Dieterich M., Sand P.K., Goldberg R.P. (2006). Successful treatment of Zoon's vulvitis with high potency topical steroid. Int Urogynecol J.

[bib0008] Brix W.K., Nassau S.R., Patterson J.W., Cousar J.B., Wick M.R. (2010). Idiopathic lymphoplasmacellular mucositis-dermatitis. J Cutan Pathol.

[bib0009] Çelik A., Haliloǧlu B., Tanriöver Y., Ilter E., Gündüz T., Ulu I. (2012). Plasma cell vulvitis: A vulvar itching dilemma. Indian J Dermatol Venereol Leprol.

[bib0010] Chan M.P., Zimarowski M.J. (2015). Vulvar dermatoses: A histopathologic review and classification of 183 cases. J Cutan Pathol.

[bib0011] Corazza M., Toni G., Virgili A., Borghi A. (2018). Plasma cell vulvitis: Further confirmation of the diagnostic utility of dermoscopy. Int J Dermatol.

[bib0012] Damiani L., Quadros M.de, Posser V., Minotto R., Boff A.L. (2017). Zoon vulvitis. An Bras Dermatol.

[bib0013] David L., Massey K. (2003). Plasma cell vulvitis and response to topical steroids: A case report. Int J STD AIDS.

[bib0014] Davis J., Shapiro L., Baral J. (1983). Vulvitis circumscripta plasmacellularis. J Am Acad Dermatol.

[bib0015] Dos Reis H.L.B., De Vargas P.R.M., Lucas E., Camporez T., De Carvalho Ferreira D. (2013). Zoon vulvitis as a differential diagnosis in an HIV-infected patient: A short report. J Int Assoc Provid AIDS Care.

[bib0016] Ee H.L., Yosipovitch G., Chan R., Ong B.H., Ee M. (2003). Resolution of vulvitis circumscripta plasmacellularis with topical imiquimod: Two case reports. Br J Dermatol.

[bib0017] Elghobashy M., Elgaly M., Salmons N., El-Ghobashy A. (2020). Hypertrophic herpes simplex with subsequent development of plasma cell vulvitis: A potential diagnostic pitfall. Int J Gynecol Pathol.

[bib0018] Fernández-Aceñero M.J., Córdova S. (2010). Zoon's vulvitis (vulvitis circumscripta plasmacellularis). Arch Gynecol Obstet.

[bib0019] Frega A., Rech F., French D. (2006). Imiquimod treatment of vulvitis circumscripta plasmacellularis. Int J Gynecol Obstet.

[bib0020] Greco A., Marinelli C., Fusconi M., Macri G.F., Gallo A., De Virgilio A. (2016). Clinic manifestations in granulomatosis with polyangiitis. Int J Immunopathol Pharmacol.

[bib0021] Gunter J., Golitz L. (2005). Topical misoprostol therapy for plasma cell vulvitis: A case series. J Low Genit Tract Dis.

[bib0022] Gurumurthy M., Cairns M., Cruickshank M. (2010). Case series of zoon vulvitis. J Low Genit Tract Dis.

[bib0023] Hautmann G., Geti V., Difonzo E.M. (1994). Vulvitis circumscripta plasmacellularis. Int J Dermatol.

[bib0024] Hindle E., Yell J., Andrew S., Tasker M. (2006). Plasma cell vulvovaginitis - A further case. J Obstet Gynaecol (Lahore).

[bib0025] Jaimes Suarez J., Vidal Conde L., Collazos Robles R., Grande Gomez J., Martin Díaz V., Parra Rodriguez O. (2017). Zoon vulvitis treated successfully with platelet-rich plasma: First case reported. J Low Genit Tract Dis.

[bib0026] Kavanagh G.M., Burton P.A., Kennedy C.T.C. (1993). Vulvitis chronica plasmacellularis (Zoon's vulvitis). Br J Dermatol.

[bib0027] Kuniyuki S., Asada T., Yasumoto R. (1998). A case of vulvitis circumscripta plasmacellularis positive for herpes simplex type II antigen. Clin Exp Dermatol.

[bib0028] McCreedy C.A., Melski J.W. (1990). Vulvar erythema. Vulvitis chronica plasmacellularis (Zoon's vulvitis). Arch Dermatol.

[bib0029] Mitchell L.S., Barela K.M., Krapf J., Govind V., Tolson H.T., Goldstein A. (2020). Plasma cell vaginitis and cervicitis. J Case Reports Images Obstet Gynecol.

[bib0030] Morioka S., Nakajima S., Yaguchi H., Naito K., Iwahara K., Ogawa H. (1988). Vulvitis circumscripta plasmacellularis treated successfully with interferon alpha. J Am Acad Dermatol.

[bib0031] Nedwich J.A., Chong K.C. (1987). Zoon's vulvitis. Australas J Dermatol.

[bib0032] Neri I., Patrizi A., Marzaduri S., Marini R., Negosanti M. (1995). Vulvitis plasmacellularis: Two new cases. Genitourin Med.

[bib0033] Page S.S., Hospital R.P., Tait C., Hospital R.P., Dykstra C. (2015). Plasma cell vulvitis: A case series. J Am Acad Dermatol.

[bib0034] Pourzan G., Laird-Fick H.S. (2019). A vexing vulvitis: A case report and review of plasma cell vulvitis. J Obstet Gynaecol (Lahore).

[bib0035] Powers R., States U., Zinn Z., States U., Kovach R., States U. (2015). Plasma cell vulvitis occurring in preexisting lichen sclerosus. J Am Acad Dermatol.

[bib0036] Robinson J.B., Im D.D., Simmons-O'Brien E., Rosenshein N.B. (1998). Etretinate: Therapy for plasma cell vulvitis. Obstet Gynecol.

[bib0037] Salopek T.G., Siminoski K. (1996). Vulvitis circumscripta plasmacellularis (Zoon's vulvitis) associated with autoimmune polyglandular endocrine failure. Br J Dermatol.

[bib0038] Santonja C., Suárez-Peñaranda J.M., Carrasco L., Fariña M.D.C., Requena L. (2019). Mucinous metaplasia of the vulva in Zoon vulvitis and lichen sclerosus et atrophicus. Description of 3 additional cases of a rarely reported histopathologic finding. Am J Dermatopathol.

[bib0039] Scurry J., Dennerstein G., Brenan J., Ostor A., Mason G., Dorevitch A. (1993). Vulvitis circumscripta plasmacellularis. A clinicopathologic entity?. J Reprod Med.

[bib0040] Sharma B., Jain V., Narang T., Radotra B.D. (2019). Plasma cell vulvitis presenting as postmenopausal atrophic vaginitis - A case report. J Obstet Gynaecol.

[bib0041] Solt I., Lowenstein L., Amit A., Bergman R., Kerner H. (2004). Ulcerative vulvitis circumscripta plasmacellularis. Isr Med Assoc J.

[bib0042] Souteyrand P., Wong E., MacDonald D.M. (1981). Zoon's balanitis (Balanitis circumscripta plasmacellularis). Br J Dermatol.

[bib0043] Thomson M.A., Carr R.A., Ganesan R., Humphreys F. (2007). Extensive mucinous metaplasia of the vulva arising within Zoon's vulvitis. Br J Dermatol.

[bib0044] Toeima E., Sule M., Warren R., Igali L. (2011). Diagnosis and treatment of Zoon's vulvitis. J Obstet Gynaecol.

[bib0045] Van Der Meijden W.I., Ewing P.C. (2011). Papular colpitis: A distinct clinical entity?: Symptoms, signs, histopathological diagnosis, and treatment in a series of patients seen at the Rotterdam Vulvar Clinic. J Low Genit Tract Dis.

[bib0046] van Kessel M.A., van Lingen R.G., Bovenschen H.J. (2010). Vulvitis plasmacellularis circumscripta in pre-existing lichen sclerosus: Treatment with imiquimod 5% cream. J Am Acad Dermatol.

[bib0047] Virgili A., Levratti A., Zampino M.R., Corazza M. (2004). Are patch tests useful in plasma cell vulvitis?. J Reprod Med Obstet Gynecol.

[bib0048] Virgili A., Levratti A., Marzola A., Corazza M. (2005). Retrospective histopathologic reevaluation of 18 cases of plasma cell vulvitis. J Reprod Med Obstet Gynecol.

[bib0049] Virgili A., Mantovani L., Lauriola M.M., Marzola A., Corazza M. (2008). Tacrolimus 0.1% ointment: Is it really effective in plasma cell vulvitis? Report of four cases. Dermatology.

[bib0050] Virgili A., Borghi A., Minghetti S., Corazza M. (2015). Comparative study on topical immunomodulatory and anti-inflammatory treatments for plasma cell vulvitis: Long-term efficacy and safety. J Eur Acad Dermatology Venereol.

[bib0051] Virgili A., Corazza M., Minghetti S., Borghi A. (2015). Symptoms in plasma cell vulvitis: First observational cohort study on type, frequency and severity. Dermatology.

[bib0052] Woodruff J.D., Sussman J., Shakfeh S. (1989). Vulvitis circumscripta plasmocellularis: A report of four cases. J Reprod Med Obstet Gynecol.

[bib0053] Yoganathan S., Bohl T.G., Mason G. (1994). Plasma cell balanitis and vulvitis (of Zoon). A study of 10 cases. J Reprod Med.

[bib0054] Zoon J.J. (1952). Differential diagnosis between chronic circumscribed benign plasmacellular balanitis and Queyrat's erythroplasia. Ned Tijdschr Geneeskd.

